# Bevacizumab for refractory gastrointestinal angiodysplasia: a case report and literature review

**DOI:** 10.1097/MEG.0000000000003059

**Published:** 2025-08-29

**Authors:** Alicia Furumaya, Lia C.M.J. Goltstein, Maarten E. Tushuizen, Michael Klemt-Kropp

**Affiliations:** a Department of Gastroenterology and Hepatology, Noordwest Ziekenhuisgroep, Alkmaar; b Department of Gastroenterology and Hepatology, Amsterdam Gastroenterology Endocrinology Metabolism, Amsterdam UMC, University of Amsterdam, Amsterdam; c Department of Gastroenterology and Hepatology, Radboud University Medical Center, Nijmegen; d Department of Gastroenterology and Hepatology, Leids Universitair Medisch Centrum, Leiden, The Netherlands

**Keywords:** angiodysplasia, bevacizumab, case report

## Abstract

Currently, symptomatic gastrointestinal (GI) angiodysplasia is treated with argon plasma coagulation (APC) via endoscopic procedures, supplemented with octreotide or thalidomide treatment. However, suboptimal response and side effects are often seen. Bevacizumab, an angiogenesis inhibitor, may provide an alternative systemic therapy for patients with refractory GI angiodysplasia. A 75-year-old male patient with cirrhosis and portal hypertension due to metabolic dysfunction-associated steatotic liver disease presented with recurrent anemia and overt GI bleeding. Initial endoscopic findings showed a combination of portal hypertensive gastropathy and GI angiodysplasia. Anemia persisted despite repeated APC and octreotide. After transjugular intrahepatic portosystemic shunt, portal hypertensive gastropathy resolved; however, GI angiodysplasia remained and caused refractory symptomatic anemia and overt bleeding. Finally, we resorted to off-label bevacizumab in the absence of other viable treatment options. The patient initially responded to treatment but has needed top-up dosing, the effect of which remains to be evaluated. In conclusion, we describe our initial experience with off-label bevacizumab in the treatment of refractory GI angiodysplasia. Based on our experience and literature, bevacizumab may be a viable option for patients with refractory GI angiodysplasia, which should be further evaluated in future studies before it can be implemented in clinical practice.

## Introduction

Angiodysplasias are vascular malformations of the gastrointestinal (GI) tract, responsible for up to 5% of cases of all GI bleeding [1,2]. They are characterized by tortuous and ectatic blood vessels in the submucosa or mucosa, lined by endothelium without smooth muscle. The pathophysiology of angiodysplasia is multifactorial and includes obstruction of submucosal veins, mucosal ischemia leading to angiogenesis, and abnormalities in platelet adhesion and aggregation [3]. Risk factors for the development of angiodysplasia include older age (>60 years of age), cardiovascular disease and aortic stenosis, chronic kidney disease, liver cirrhosis, and diabetes [1,4].

Patients with angiodysplasia often present with iron-deficiency anemia due to occult blood loss or with overt GI bleeding requiring transfusions and hospital admissions, significantly impacting quality of life [5]. Most patients have multiple angiodysplasias, and the majority (>60%) of angiodysplasias causing obscure overt blood loss are localized in the duodenum and jejunum [6]. Small bowel angiodysplasias can be challenging to diagnose, but can be found on extended esophagogastroscopy with a pediatric colonoscope, capsule endoscopy, or double or single balloon enteroscopy.

Endoscopic treatment by argon plasma coagulation (APC) is considered first-line treatment for symptomatic angiodysplasia. However, recurrence rates exceeding 40%, and up to 80% in the small bowel, have been reported [7]. Recurrence can be caused by angiodysplasias not initially detected or treated during the index endoscopy, or by the neoformation of angiodysplasias. Therefore, systemic pharmacological treatment may be necessary [8].

Octreotide or thalidomide has shown promising results in randomized clinical trials, leading to a reduction of transfusion units and the number of bleeding episodes. However, suboptimal response (less than 50% reduction of bleeding episodes or transfusion requirement) and side effects were frequent with both treatments (approximately 40 and 65%, respectively) [9–12]. Octreotide mainly shows cholecystolithiasis and glucose dysregulation requiring adjustments in glucose-lowering therapies [10]. Thalidomide may induce neuropathy, and its availability in certain countries may be limited [11].

Bevacizumab is a humanized mAb targeting vascular endothelial growth factor, a key regulator of angiogenesis [13]. Bevacizumab is commonly used in oncology, for example, metastatic colorectal cancer and nonsmall cell lung cancer [14–16]. Outside of the oncology field, bevacizumab is increasingly studied and used off-label in the context of hereditary hemorrhagic telangiectasia (HHT) [17,18]. Initially, success was shown in the decreasing cardiac output and reduction of epistaxis of patients with HHT [19]. Currently, phase 2 and 3 trials are ongoing (NCT04404881 and NCT03227263), and there is increasing evidence of the efficacy of bevacizumab on GI bleeding in the context of HHT [19–21].

Due to the morphological similarities between angiodysplasia and the GI manifestations of HHT, bevacizumab is being explored as a potential treatment for angiodysplasia [22]. We describe the case of a patient with liver cirrhosis and recurrent GI bleeding, who received off-label bevacizumab therapy to treat refractory angiodysplasia. In addition, we review the present literature on bevacizumab as treatment for refractory GI angiodysplasia.

## Case presentation

A 75-year-old male patient first presented in 2018 with microcytic anemia, at which time he had no macroscopic blood loss. The patient had a history of type 2 diabetes, angina pectoris, and cirrhosis caused by metabolic dysfunction-associated steatotic liver disease with signs of portal hypertension. He did not use any anticoagulative medication. Gastroscopy showed esophageal varices without signs of bleeding, for which propranolol 80 mg was started twice daily. Three small adenomatous polyps were removed at colonoscopy.

**Fig. 1.**
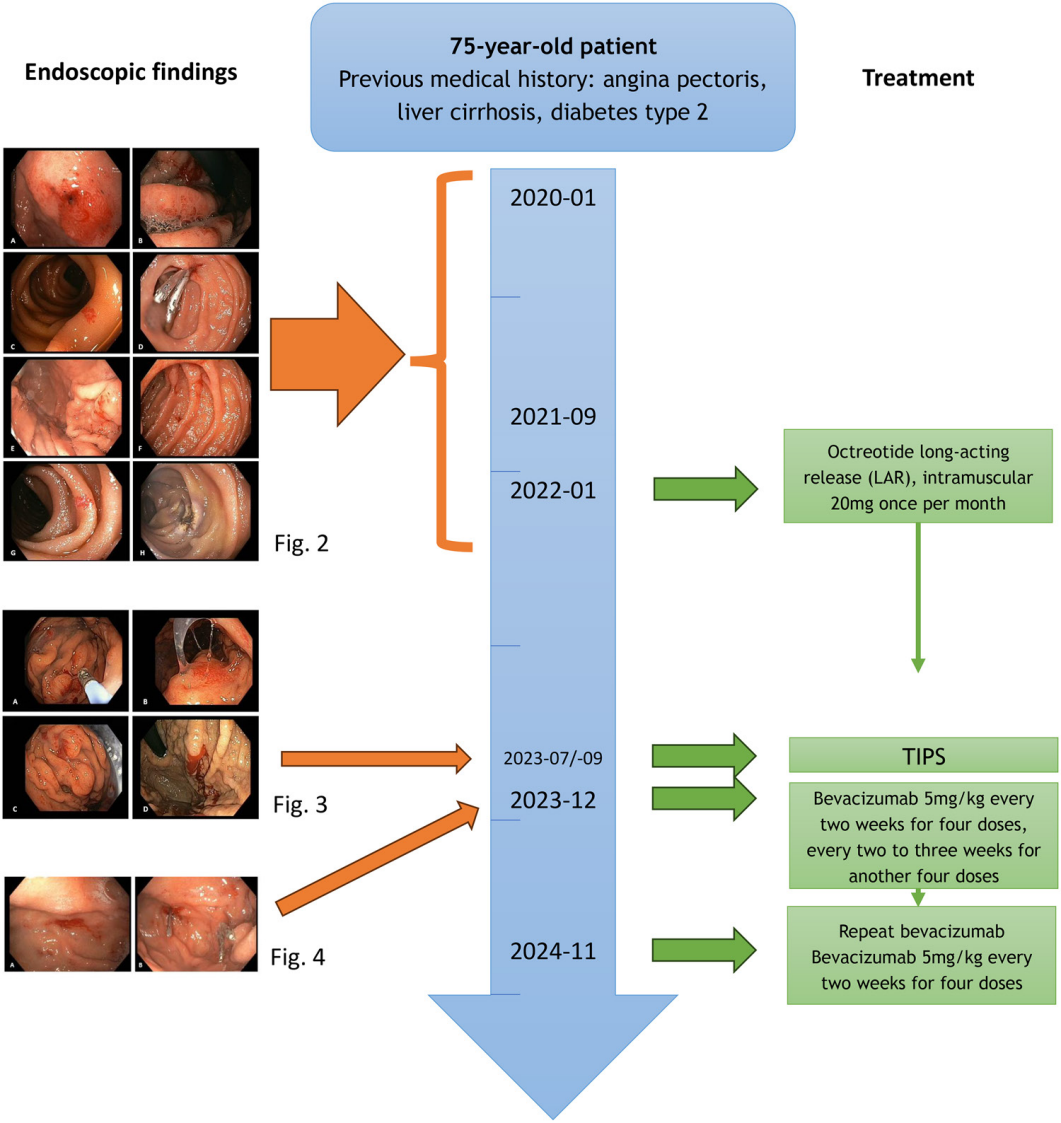
Timeline of the most relevant presentations, endoscopic findings, and treatment. Between January 2020 and January 2022, endoscopic findings were characterized by a combination of angiodysplasias and portal hypertensive gastropathy. Repeat argon plasma coagulation was performed and octreotide was started. From July to September 2023, an episode of deep and recurrent anemia occurred, mostly related to portal hypertensive gastropathy and treated by TIPS. From December 2023 onward, refractory angiodysplasia caused melena en anemia and bevacizumab was commenced. Detailed images are available in Figs. 2–4. TIPS, transjugular intrahepatic portosystemic shunt.

Between 2020 and 2024, the patient developed intermittent melena and recurrent microcytic anemia, requiring multiple blood transfusions, hospital admissions, and repeated diagnostic and interventional endoscopy. During these years, the patient was admitted twice for decompensated liver cirrhosis. The first admission was for ascites, the second admission was due to variceal bleeding, which was treated with rubber band ligation. Various potential sources of bleeding were found and treated endoscopically. Figure 1 shows a timeline of the most relevant admissions/episodes, endoscopic findings, and treatment.

**Fig. 2.**
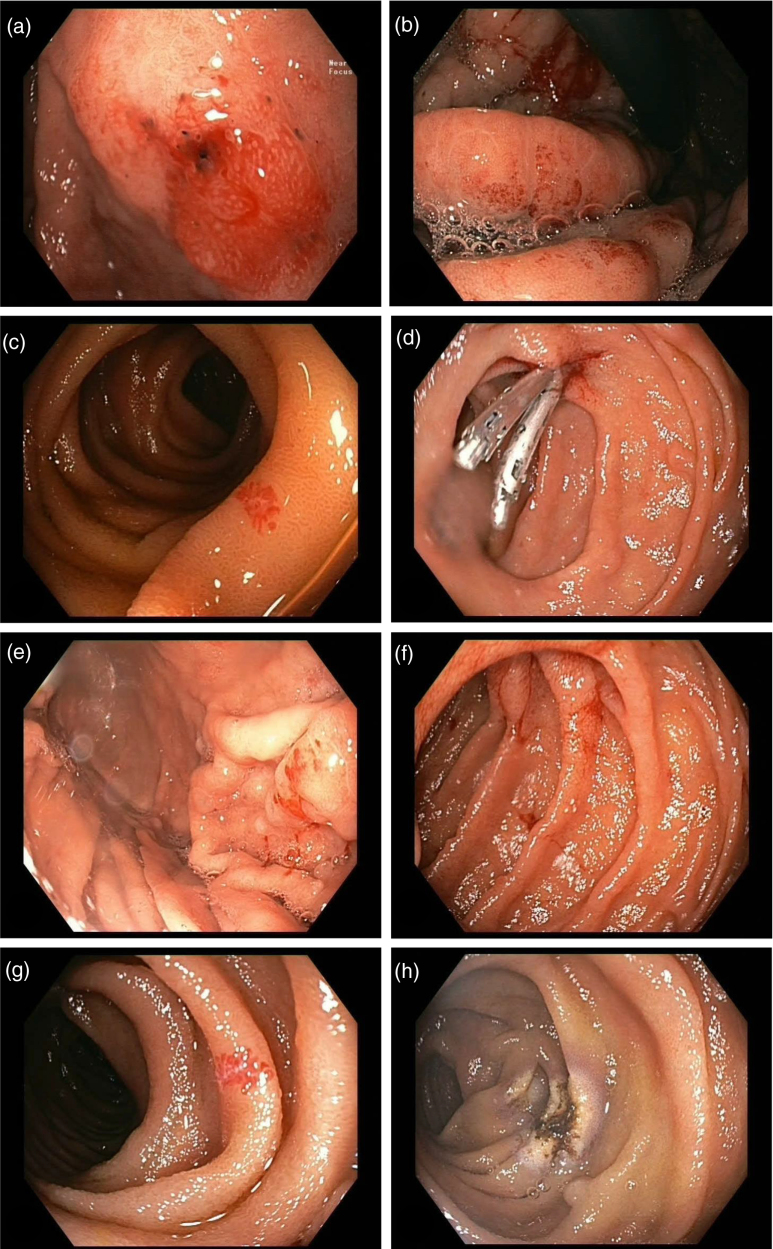
Gastroscopy images from January 2020 (a), September 2021 (b–d), and January 2022 (e–h). Angiodysplasia (c, f, and g) is characterized by their tortuous blood vessels and treated with clips and APC (d and h), respectively. A more diffuse and mosaic appearance is a classical appearance of portal hypertensive gastropathy (a, b, and e). APC, argon plasma coagulation.

Initially, in January 2020, only angiodysplasias were seen (Fig. 2a) for which APC treatment was performed. In September 2021, push enteroscopy showed portal hypertensive gastropathy (Fig. 2b) and small intestinal angiodysplasias (Fig. 2c), the latter of which were treated with APC. Due to bleeding after APC, clipping was performed as rescue therapy (Fig. 2d). In addition, octreotide long-acting release was started intramuscularly at 20 mg once per month. In January 2022, a combination of portal hypertensive gastropathy (Fig. 2e) and small bowel angiodysplasias (Figs. 2f, g) was again observed and treated with repeat APC. Octreotide was halted because the benefit of octreotide did not outweigh the side effects experienced by the patient, that is, pain at the injection site and abdominal pain.

**Fig. 3.**
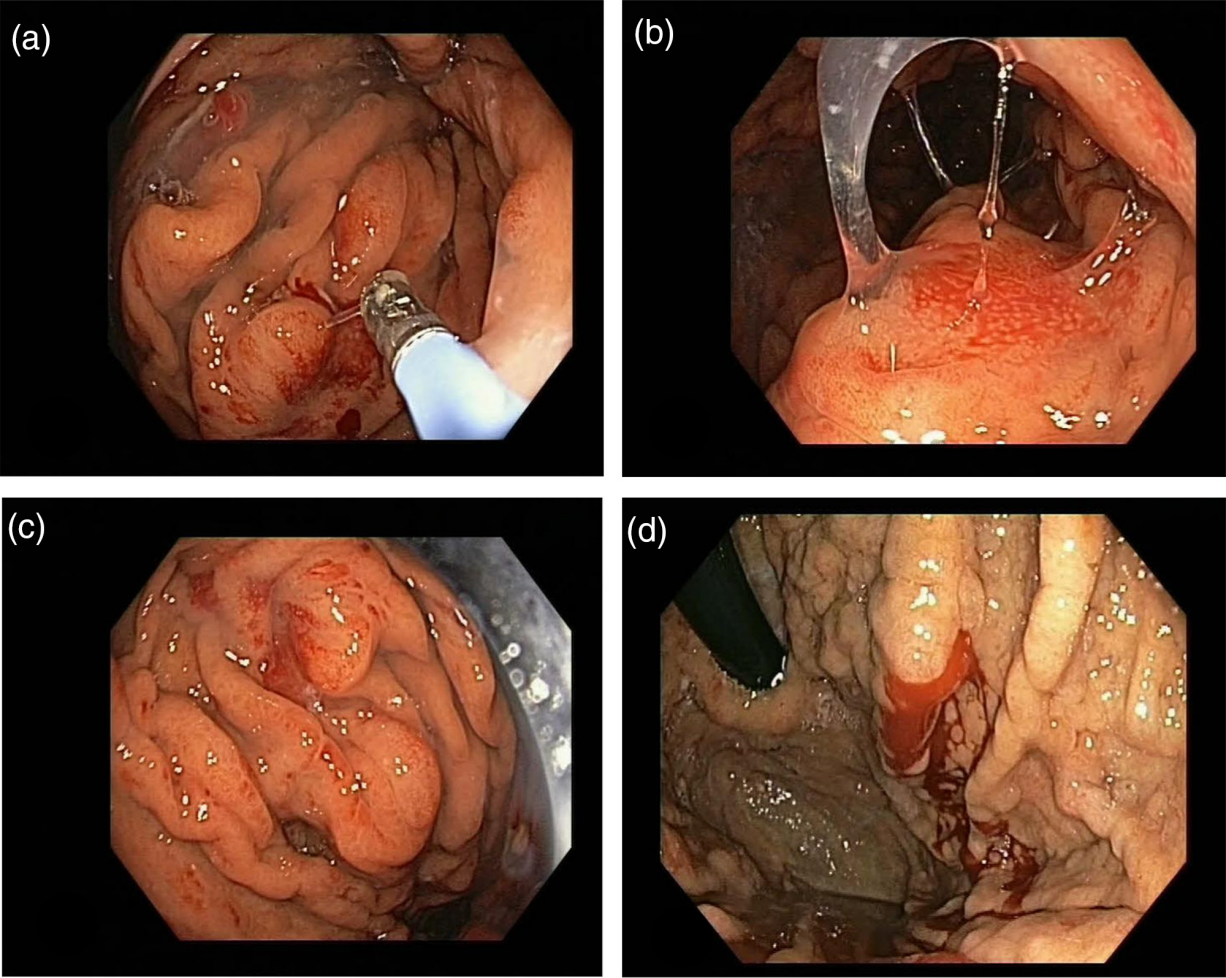
Gastroscopy between July (d) and September (a–c) 2023, showing portal hypertensive gastropathy, characterized by their diffuse and mosaic patterns.

Between July and September 2023, the patient was repeatedly admitted with deep and recurrent anemia. Gastroscopy showed signs of portal hypertensive gastropathy (Fig. 3). Capsule endoscopy showed nonbleeding intestinal angiodysplasia. At colonoscopy, three other polyps were removed, which were an unlikely source of the anemia. We then resorted to transjugular intrahepatic portosystemic shunt (TIPS) to treat the portal hypertensive gastropathy. Hepatic venous pressure gradient was 18 mmHg, confirming portal hypertension.

**Fig. 4.**
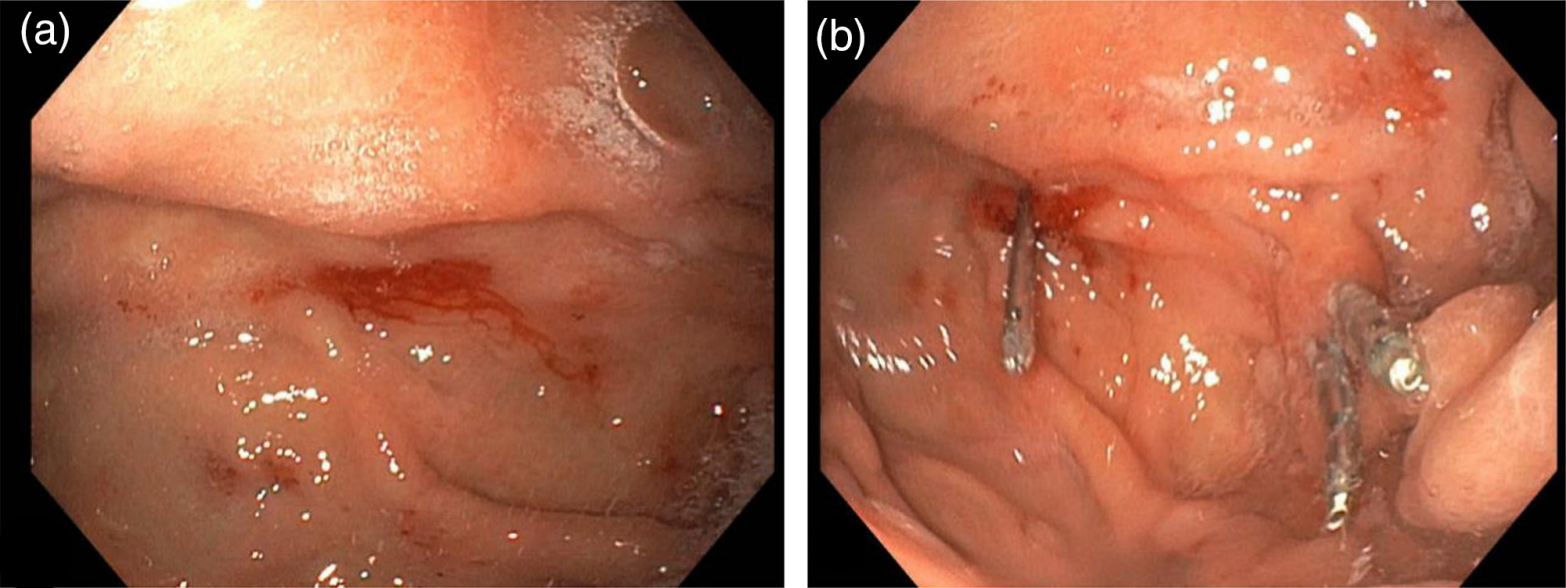
Push enteroscopy in December 2023, showing the spider-like appearance of angiodysplasia (a) and treated with clips (b).

Several weeks after TIPS, in December 2023, the patient was readmitted with melena and anemia. Initial gastroscopy and colonoscopy did not identify the source of the bleeding. During the same admission, push enteroscopy showed two intestinal angiodysplasias with bleeding tendency, which were endoscopically treated (Fig. 4). We decided not to apply thalidomide due to the comorbidity (liver cirrhosis and diabetes) of the patient. We discussed the potential risks and benefits of bevacizumab with the patient, weighing the paucity of high-level clinical evidence with the lack of other viable treatment options. Upon agreement with the patient and after approval of the insurance company, bevacizumab therapy was commenced at 5 mg/kg every 2 weeks for 4 doses. The initial dosing cycle was followed by 4 additional doses of 5 mg/kg every 2 to 3 weeks [22,23]. Hemoglobin levels increased to >8.5 mmol/l (>13.7 g/dl), which had not been within normal range since 2018.

In November 2024, 5 months after completing the second course of bevacizumab, the patient was admitted again with symptomatic anemia. Initial push enteroscopy demonstrated three nonbleeding intestinal angiodysplasias. The TIPS was patent; therefore, an expectative course was followed. However, melena persisted; therefore, bevacizumab therapy was recommenced. The patient was discharged and will continue bevacizumab therapy at 5 mg/kg every 2 weeks for another four doses. The long-term evaluation of the effect of the top-up dosing remains to be evaluated.

In total, the patient has been admitted more than 15 times within 4 years (of which two admissions exceeded a month), underwent 26 gastroscopies, four colonoscopies, two single balloon enteroscopies, and two capsule endoscopies. He received almost a hundred blood transfusions.

## Discussion

We describe our experience in the treatment of a patient with refractory angiodysplasia with off-label bevacizumab. Our patient showed no side effects and an initial, but not sustained, response. Current systemic pharmacological treatment of these patients consists of octreotide and thalidomide. In our patient, anemia recurred despite octreotide treatment. We decided not to apply thalidomide due to the comorbidity (liver cirrhosis and diabetes) of the patient. Randomized clinical trials on thalidomide showed a reduction in bleeding in patients with small bowel angiodysplasia. These studies excluded patients with insulin-dependent diabetes [11]. Moreover, thalidomide might lead to hepatic encephalopathy, especially if applied after TIPS, which already increases the risk of hepatic encephalopathy [24,25]. In the context of HHT, there is anecdotal evidence that bevacizumab may be more effective than thalidomide [18]. Nonetheless, compared to octreotide and bevacizumab, thalidomide is a less expensive alternative.

In our patient, the comorbid disease of liver cirrhosis with portal hypertension and type 2 diabetes provided a diagnostic and therapeutic challenge. First, the patient showed signs of both portal hypertensive gastropathy and angiodysplasia. The nonresponse of our patient to TIPS may suggest that the anemia and bleeding were due to angiodysplasia rather than portal hypertensive gastropathy. However, portal hypertensive gastropathy is an important, often occurring differential diagnosis in patients with recurrent melena and anemia and portal hypertension.

A literature review (Supplementary Digital Content, Supplemental Digital Content 1, https://links.lww.com/EJGH/B209) identified only one retrospective cohort study and a few case reports describing the use of bevacizumab in angiodysplasia [21,22,26–28]. In the cohort study by Albitar *et al.* [22], 21 patients with refractory bleeding and transfusion-dependent anemia due angiodysplasia were included. The need for endoscopic procedures and red blood cell transfusions decreased significantly after 12 months (median 2 to 0, *P* = 0.0029 and median 20 to 2, *P* < 0.0001). It is important to note that there are doubts about the safety of bevacizumab in patients with portal hypertension. Bevacizumab may increase the risk of portal hypertension-related GI bleeding and therefore it might be necessary to control portal hypertension first [29,30]. Therefore, the use of bevacizumab should be carefully considered, especially in the presence of liver cirrhosis and portal hypertension.

Top-up dosing of bevacizumab may be necessary in more than 60% of patients, in line with findings in HHT [22]. Monthly laboratory testing was recommended in all patients by Albitar *et al.*, in order to recommend timely top-up dosing. In our patient, hemoglobin levels were stable in the last routine assessment, a month before the patient was readmitted with recurrent anemia.

In conclusion, current therapies for angiodysplasia are limited to endoscopic treatment with octreotide and thalidomide. Bevacizumab might be a promising pharmacological addition or alternative. However, further studies are needed to evaluate the effect and the cost-efficacy of bevacizumab for refractory angiodysplasia.

## Acknowledgements

### Conflicts of interest

There are no conflicts of interest.

## Supplementary Material


