# Supplementary material for: Bevacizumab for refractory gastrointestinal angiodysplasia: a case report and literature review
Source: Eur J Gastroenterol Hepatol. 2025 Aug 29;37(12):1396–401. doi: 10.1097/MEG.0000000000003059 (PMC12577662; doi:10.1097/MEG.0000000000003059)
Supplement: Supplementary file 1 [file ejgh-37-1396-s001.docx]

**Supplementary Digital Content (SDC)**

**Literature review strategy**

Search criteria:

(Bevacizumab[tiab] OR bevacizumab[MeSH]) AND ((angioectasia*[tiab] OR Ectasia*[tiab] OR angiodysplasia*[tiab]) AND (bowel[tiab] OR gastr*[tiab] OR antral[tiab] OR intestinal[tiab]) OR GAVE[tiab] OR GVE[tiab] OR angiodysplasia[MeSH])

Searched on 10/02/2025

Inclusion criteria: case reports, cohort study and randomized clinical trials including adult patients undergoing bevacizumab treatment for refractory gastrointestinal angiodysplasia

Exclusion criteria:

- Review articles
- Studies including patients undergoing bevacizumab treatment for other indications (such as macular degeneration, malignancies)
- Studies including patient undergoing bevacizumab treatment for refractory gastrointestinal dysplasia in the context of a coagulation or genetic disorder, Heyde syndrome, left ventricular assist devices or hereditary hemorrhagic telangiectasia

Results: 66 studies
